# From Classification to Causality: Advancing Understanding of Mechanisms of Change in Implementation Science

**DOI:** 10.3389/fpubh.2018.00136

**Published:** 2018-05-07

**Authors:** Cara C. Lewis, Predrag Klasnja, Byron J. Powell, Aaron R. Lyon, Leah Tuzzio, Salene Jones, Callie Walsh-Bailey, Bryan Weiner

**Affiliations:** ^1^Kaiser Permanente Washington Health Research Institute, Seattle, WA, United States; ^2^Department of Psychological and Brain Sciences, Indiana University, Bloomington, IN, United States; ^3^Department of Psychiatry and Behavioral Sciences, University of Washington, Seattle, WA, United States; ^4^Department of Health Policy and Management, Gillings School of Global Public Health, University of North Carolina at Chapel Hill, Chapel Hill, NC, United States; ^5^Public Health Sciences Division, Fred Hutchinson Cancer Research Center, Seattle, WA, United States; ^6^Department of Global Health, University of Washington, Seattle, WA, United States

**Keywords:** implementation, mechanism, mediator, moderator, theory, causal pathway, strategy

## Abstract

**Background:**

The science of implementation has offered little toward understanding *how* different implementation strategies work. To improve outcomes of implementation efforts, the field needs precise, testable theories that describe the causal pathways through which implementation strategies function. In this perspective piece, we describe a four-step approach to developing causal pathway models for implementation strategies.

**Building causal models:**

First, it is important to ensure that implementation strategies are appropriately specified. Some strategies in published compilations are well defined but may not be specified in terms of its core component that can have a reliable and measureable impact. Second, linkages between strategies and mechanisms need to be generated. Existing compilations do not offer mechanisms by which strategies act, or the processes or events through which an implementation strategy operates to affect desired implementation outcomes. Third, it is critical to identify proximal and distal outcomes the strategy is theorized to impact, with the former being direct, measurable products of the strategy and the latter being one of eight implementation outcomes (1). Finally, articulating effect modifiers, like preconditions and moderators, allow for an understanding of where, when, and why strategies have an effect on outcomes of interest.

**Future directions:**

We argue for greater precision in use of terms for factors implicated in implementation processes; development of guidelines for selecting research design and study plans that account for practical constructs and allow for the study of mechanisms; psychometrically strong and pragmatic measures of mechanisms; and more robust curation of evidence for knowledge transfer and use.

## Background: Why Build Causal Pathway Models?

In recent years, there has been growing recognition of the importance of implementing evidence-based practices as a way to improve the quality of health care and public health. However, the results of implementation efforts have been mixed. About two-thirds of efforts fail to achieve the intended change (2), and nearly half have no effect on outcomes of interest (3). Implementation strategies are often mismatched to barriers [e.g., training, a strategy that could affect implementation outcomes through changes in an individual’s knowledge (intrapersonal-level), is used inappropriately to address an organizational-level barrier like poor culture] (4), and implementation efforts are increasingly complex and costly without enhanced impact (5). These suboptimal outcomes are due, in large part, to the dearth of tested theory in the field of implementation science (6). In particular, the field has a limited understanding of *how* different implementation strategies work—the specific causal mechanisms through which implementation strategies influence care delivery [7; Lewis et al. (under review)^1^]. As a consequence, implementation science has been limited in its ability to effectively inform implementation practice by providing guidance about when and in what contexts specific implementation strategies should be used and, just as importantly, when they should not.

The National Academy of Science defines “science” as “the use of evidence to construct testable explanations and predictions of natural phenomena, as well as the knowledge generated through this process.” (8) The field of implementation has spent the past two decades building and organizing knowledge, but we are far from having testable explanations that afford us the ability to generate predictions. To improve outcomes of implementation efforts, the field needs testable theories that describe the causal pathways through which implementation strategies function (6, 9). Unlike frameworks, which offer a basic conceptual structure underlying a system or concept (10), theories provide a testable way of explaining phenomena by specifying relations among variables, thus enabling prediction of outcomes (10, 11).

Causal pathway models represent interrelations among variables and outcomes of interest in a given context (i.e., the building blocks of implementation theory). Specifying the structure of causal relations enables scientists to empirically test whether the implementation strategies are operating *via* theorized mechanisms, how contextual factors moderate the causal processes through which implementation strategies operate, and how much variance in outcomes is accounted for by those mechanisms. Findings from studies based on causal models can, over time, both help the field develop more robust theories about implementation processes and advance the practice of implementation by addressing key issues. For instance, causal models can do the following: (1) inform the development of improved implementation strategies, (2) identify mutable targets for new strategies, (3) increase the impact of existing strategies, and (4) prioritize which strategies to use in which contexts.

In this perspective piece, we propose an approach to theory development by specifying, in the form of causal pathway models, hypotheses about the causal operation of different implementation strategies in various settings, so that these hypotheses can be tested and refined. Specifically, we offer a four-step process to developing causal pathway models for implementation strategies. Toward this end, we argue the field must move beyond having lists of variables that can rightly be considered determinants [i.e., factors that obstruct or enable change in provider behavior or health-care delivery processes (12)], and toward precise articulation of mediators, moderators, preconditions, and (proximal versus distal) outcomes (see Table 1 for definitions).

**Table 1:** Terms and definitions.

Term	Definition
Mechanism	Process or event through which an implementation strategy operates to affect desired implementation outcomes

Precondition	Factor that is necessary in order for an implementation mechanism to be activated

Determinant	Also commonly referred to as “barriers” and “facilitators,” a factor that enables or hinders the implementation strategy from eliciting the desired effect

Mediator	Intervening variable that may account for the relationship between the implementation strategy and the implementation outcome

Moderator	Factor that increase or decrease the level of influence of an implementation strategy

Proximal outcome	The product of the implementation strategy that is realized because of its specific mechanism of action, the most immediate, observable outcome in the causal pathway

Distal outcome	Outcomes that the implementation processes is ultimately intended to achieve, not the most immediate outcome in the causal pathway

## Building Causal Pathway Models

**Figure 1:**
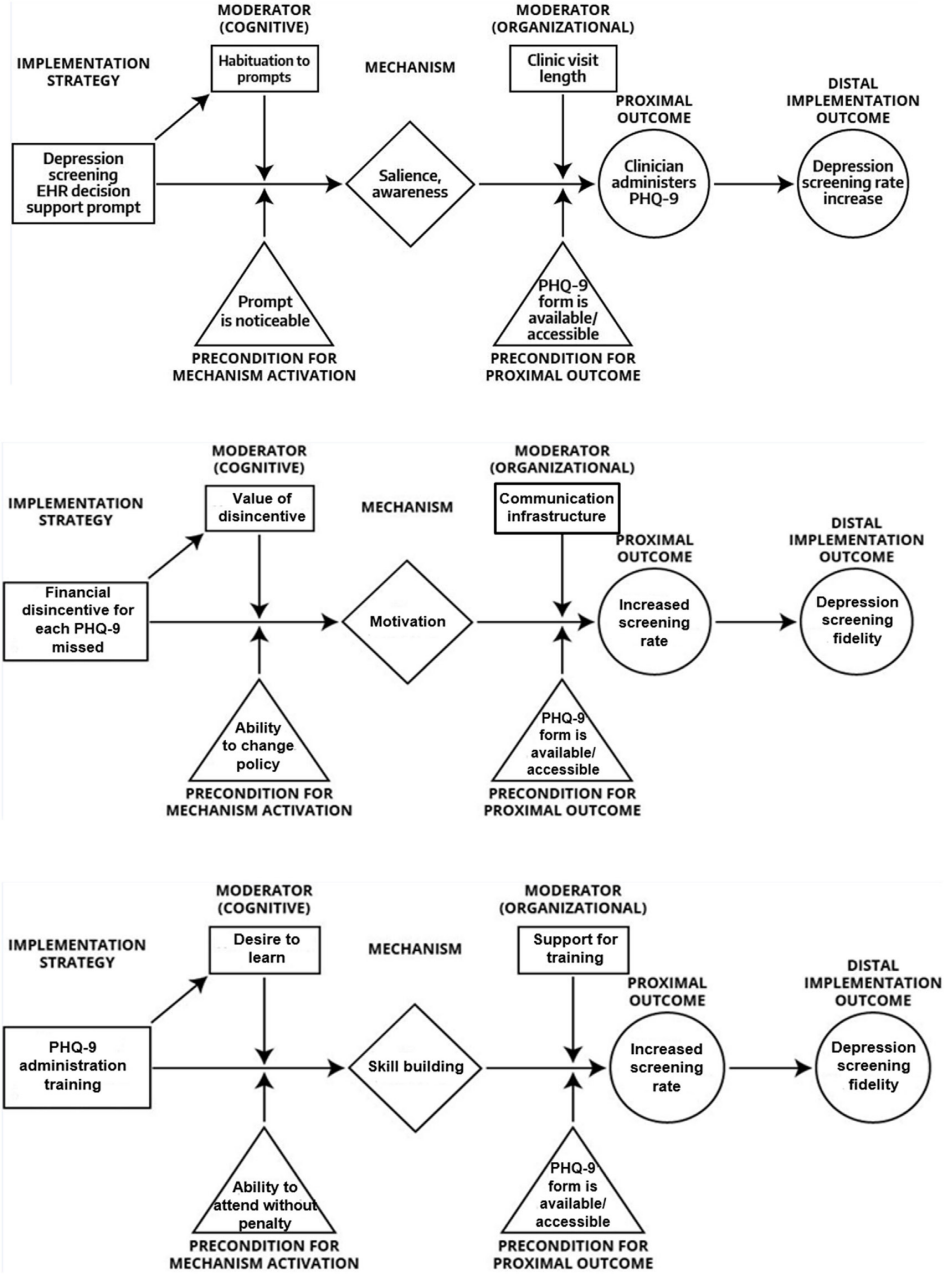
Causal model diagrams.

Our perspective draws upon Agile Science (13, 14)—a new method for developing and studying behavioral interventions that focuses on intervention modularity, causal modeling, and efficient evaluations to generate empirical evidence with clear boundary conditions (in terms of population, context, behavior, etc.) to maximize knowledge accumulation and repurposing. Agile Science has been used to investigate goal-setting interventions for physical activity, engagement strategies for mobile health applications, depression interventions for primary care, and automated dietary cues to promote weight loss (13, 15). Applied to implementation strategies, Agile Science-informed causal pathway diagram modeling consists of at least four steps: (1) specifying implementation strategies; (2) generating strategy-mechanism linkages; (3) identifying proximal and distal outcomes; and (4) articulating moderators and preconditions. To demonstrate this approach, we offer examples of causal pathway models for a set of three diverse implementation strategies (see Figure 1). The strategies are drawn from the following example. A community mental health center is planning to implement measurement-based care in which providers solicit patient-reported outcome data [e.g., Patient Health Questionnaire 9-item depression symptom severity measure (16)] prior to clinical encounters to inform treatment (17). The community mental health center plans to use training, financial penalty (disincentives), and audit and feedback as they are common strategies used to support measurement-based care implementation (18).

### Step 1: Specifying Implementation Strategies

The Expert Recommendations for Implementing Change study yielded a compilation of 73 implementation strategies (19) developed by a multidisciplinary team through a structured literature review (20), Delphi process, and concept mapping exercise (19, 21, 22). Thus, there exists a solid foundation of strategies that are conceptually clear and well defined. However, the compilation was never explicitly linked to mechanisms. Following Kazdin (7), we define “mechanisms” as the processes or events through which an implementation strategy operates to effect desired implementation outcomes. Upon careful examination, it seems many strategies are not well enough specified to be linked to mechanisms in a coherent manner, a key step in causal model building. For instance, the compilation of 73 strategies lists “learning collaboratives,” a general approach for which the discrete strategies or core components are underspecified. This makes it difficult to identify their precise mechanisms of action (23). Underspecified strategies also leave the field vulnerable to inappropriately synthesizing data across studies (24, 25).

In our case example, training is a strategy that is underspecified. We adapted procedures from Michie et al. (26) to guide strategy specification recommending that each strategy be assessed for whether it: (1) aims to promote the adoption, implementation, sustainment, or scale-up of an evidence-based practice; (2) is a proposed “active ingredient” of adoption, implementation, sustainment, or scale-up; (3) represents the smallest component while retaining the proposed active ingredient; (4) can be used alone or in combination with other discrete strategies; (5) is observable and replicable; and (6) can have a measureable impact on specified mechanisms of implementation (and, if so, whether putative mechanisms can be listed). If strategies do not meet these criteria, they require revision and further specification. This could involve suggesting alternative definitions, eliminating an implementation strategy altogether, or articulating a new, narrower strategy that is a component or a type of the original strategy. Training would meet all but the third and sixth criteria (listed previously), because training can be comprised of several active ingredients (e.g., didactics, modeling, role play/rehearsal, feedback, shadowing) each of which may operate on an unique mechanism. In this case, training ought to be more narrowly defined to make clear its core components.

### Step 2: Generating Strategy-Mechanism Linkages

Once specified, an implementation strategy needs to be linked to the mechanisms hypothesized to underlie its functioning. Mechanisms explain *how* an implementation strategy has an effect by describing the actions that lead from the administration of the strategy to the implementation outcomes (see Table 1 for definitions). Statistically speaking, mechanisms are always mediators, but mediators may not be mechanisms. Similarly, moderators can point toward mechanisms but are not themselves reliably mechanisms. Determinants may explain why an implementation strategy did or did not have an effect, but mechanisms explain *how* a strategy had an effect, by, for example, altering the status of a determinant. Determinants are naturally occurring, and often but not always, malleable factors that could prevent or enable the strategy to affect the desired outcomes. Mechanisms are intentionally activated by the application of an implementation strategy and can operate at different levels of analysis, such as at the levels of intrapersonal (e.g., learning), interpersonal (e.g., sharing), organizational (e.g., leading), community (e.g., restructuring), and macro policy (e.g., guiding) (27). For an implementation effort to be successful, chosen strategies should be compatible with and able to act on the local determinants [e.g., provider habit (determinant) is addressed with clinical decision support (strategy) *via* self-reflection/reflecting (mechanism)]. Although commonly used in implementation science, we propose that the notion of a determinant is insufficiently specific as researchers have used it to refer to at least two types of variables in a causal process: proximal outcomes and effect modifiers (see text footnote 1). Our discussion below uses these more precise terms instead.

Most implementation strategies likely act *via* multiple mechanisms, although it remains an empirical question whether one mechanism is primary and others are ancillary. It is also likely that the same mechanism might be involved in the operation of multiple implementation strategies. Initial assessment of strategy-mechanism linkages is made in the context of the broader scientific knowledge base about how a strategy produces an outcome (7). For instance, many strategies have their own literature base (e.g., audit and feedback) (28) that offer theoretical and empirical insights about which mechanisms might be underlying the functioning of those strategies [e.g., reflecting, learning, and engaging (28)]. Effort should always be made to draw upon and test existing theories, but if none offer sufficient guidance, hypothesizing variables that may have causal influence remains critical. In this way, over time, the initially formulated strategy-mechanism linkages can be reassessed and refined as studies begin to test them empirically. While such empirical evaluations are currently rare—across two systematic reviews of implementation mechanisms, only 31 studies were identified and no mechanisms were empirically established (see text footnote 1; 29)—the causal pathway models we propose here are explicitly intended to facilitate evaluations of the mechanistic processes through which implementation strategies operate.

### Step 3: Identifying Proximal and Distal Outcomes

Implementation scientists have isolated eight outcomes as the desired endpoints of implementation efforts: acceptability, feasibility, appropriateness, adoption, penetration, fidelity, cost, and sustainability (1). Many of these outcomes are appropriately construed as latent variables, but others are manifest/observable in nature (30); a recent systematic review offers measures of these outcomes and measure meta-data (31). In terms of the causal processes through which implementation strategies operate, these outcomes are often best conceptualized as *distal outcomes* that the implementation process is intended to achieve, and each of them may be more salient at one phase of implementation than another. For instance, with the Exploration, Preparation, Implementation, Sustainment Framework (32), acceptability of an evidence-based practice may be most salient in the exploration phase, whereas fidelity may be the goal of an implementation phase. Despite the plausible temporal interrelations among the outcomes, mounting evidence indicates that not all implementation strategies influence each of the aforementioned outcomes (e.g., workshop training can influence adoption but not fidelity) (33). To fully establish the plausibility of an implementation mechanism and a testable causal pathway, proximal outcomes must be expounded.

Proximal outcomes are direct, measurable, and typically observable, products of the implementation strategy that occur because of its specific mechanism of action. That is, affecting a proximal outcome in the intended direction can confirm/disconfirm activation of the putative mechanism, offering a low-inference way to establish evidence for a theorized mechanism. Most often, mechanisms themselves cannot be directly measured, forcing (either high-inference assessment or) reliance on the observation of change in a proximal outcome of interest. For instance, didactic education, as an active ingredient of training, acts primarily through the mechanism of learning on the proximal outcome of knowledge to influence the distal implementation outcome of perceived acceptability or even adoption. Practice with feedback acts through the mechanism of reflecting on proximal outcomes of skills and confidence to influence the distal implementation outcome of adoption or even fidelity. To identify proximal outcomes, one must answer the question, “How will I know if this implementation strategy had an effect *via* the mechanism that I think it is activating?” or “What will be different if the hypothesized mechanisms for this strategy is at play?” It is very common for mechanisms and proximal outcomes to be conflated in the literature given that researchers often test mediation models examining the impact of a strategy on a distal implementation outcome *via* a more proximal outcome. The way we are using the terms, a mechanism is a process through which an implementation strategy operates, and a proximal outcome is a measurable effect of that process that is in the causal pathway toward the distal implementation outcomes.

### Step 4: Articulating Effect Modifiers

Finally, there are two types of effect modifiers that are important to articulate, both of which can occur across multiple levels of analysis: moderators and preconditions. Moderators are factors that increase or decrease the level of influence of an implementation strategy on an outcome. See Figure 1 in which an example for intra-individual and organizational-level moderators for audit and feedback are articulated. Theoretically, moderators are factors that interact with a strategy’s mechanism of action, even if exactly how they interact mechanistically are not understood. Preconditions are factors that are necessary for an implementation mechanism to be activated at all (see Figure 1). They are necessary conditions that need to be in place for the causal process that leads from an implementation strategy to its proximal and distal outcomes to take place. Both moderators and preconditions are most often mischaracterized as “determinants” in the implementation science literature base, which may limit our ability to understand the nature of the relations between a strategy and the individual and contextual factors that modify its effects, and, in turn, where, when, and why strategies have an effect on outcomes of interest.

## Future Directions: What the Field of Implementation Needs to Fully Establish Itself as a Science

In order to fully establish itself as a science by offering testable explanations and enabling the generation of predictions, we offer four critical steps for the field of implementation: (1) specify implementation strategies; (2) generate implementation strategy-mechanism linkages; (3) identify proximal and distal outcomes; and (4) articulate effect modifiers. In addition to these steps, we suggest that future research should strive for the generation of precise terms for factors implicated in implementation processes and use them consistently across studies. In a systematic review of implementation mechanisms, researchers conflated preconditions, predictors, moderators, mediators, and proximal outcomes (see text footnote 1). In addition, there is room for the field to develop guidelines for selecting research designs and study plans that account for practical constraints of the contexts in which implementation is studied *and* allow for mechanism evaluation. The types of causal pathway models that we advocated for here, paired with an understanding of the constraints of a particular study site, would enable researchers to select appropriate methods and designs to evaluate hypothesized relations by carefully considering the temporal dynamics such as how often a mechanism should be measured and how much the outcome is expected to change and when.

In order to truly advance the field, much work needs to be done to identify or develop psychometrically strong and pragmatic measures of implementation mechanisms. Empirically evaluating causal pathway models requires psychometrically strong measures of mechanisms that are also pragmatic, yet none of the seven published reviews of implementation-relevant measures focus on mechanisms. It is likely that measure development will be necessary to advance the field. Finally, implementation science could benefit from the building of more robust curation of evidence for knowledge transfer and use. Other fields house web-based databases for collecting, organizing, and synthesizing empirical findings [e.g., Science of Behavior Change (34)]. In doing so, fields can accumulate knowledge more rapidly and users of knowledge can determine what is working, when, and why, as well as what generalizes and what does not. Such curation of evidence can more efficiently lead to the development of improved implementation strategies (e.g., through strategy specification), identification of mutable targets for new strategies (e.g., mechanisms revealed for existing strategies that may not be pragmatic), and prioritization of strategy use for a given context (e.g., given knowledge of preconditions and moderators).

## Author Contributions

CL and PK are co-first authors, who co-led manuscript development. CL and BW are co-PIs on an R01 proposal that led to the inception of this manuscript. All authors (CL, PK, BP, AL, LT, SJ, CW-B, and BW) contributed to idea development, writing, and editing of this manuscript and agreed with its content.

## Conflict of Interest Statement

The authors declare that the research was conducted in the absence of any commercial or financial relationships that could be construed as a potential conflict of interest. The reviewer TW declared a past co-authorship with one of the author BP to the handling Editor.

## Footnotes

^1^Lewis CC, Boyd MR, Walsh-Bailey C, Lyon AR, Beidas RS, Mittman B, et al. A systematic review of empirical studies examining mechanisms of dissemination and implementation in health. *Implement Sci* (under review).
